# Cancer-associated fibroblasts promote pro-tumor functions of neutrophils in pancreatic cancer via IL-8: potential suppression by pirfenidone

**DOI:** 10.1007/s00262-025-03946-z

**Published:** 2025-02-04

**Authors:** Tomohiko Yagi, Shunsuke Kagawa, Shohei Nogi, Atsuki Taniguchi, Masashi Yoshimoto, Kanto Suemori, Yasuo Nagai, Shuto Fujita, Shinji Kuroda, Satoru Kikuchi, Yoshihiko Kakiuchi, Fuminori Teraishi, Kosei Takagi, Toshiaki Ohara, Hiroshi Tazawa, Toshiyoshi Fujiwara

**Affiliations:** 1 Department of Gastroenterological Surgery, Okayama University Graduate School of Medicine, Dentistry and Pharmaceutical Sciences, 2-5-1 Shikata-Cho, Kita-Ku, Okayama, 700-8558 Japan; 2 Minimally Invasive Therapy Center, Okayama University Hospital, Okayama, Japan; 3 Center for Innovative Clinical Medicine, Okayama University Hospital, Okayama, Japan; 4 Departments of Pathology and Experimental Medicine, Okayama University Graduate School of Medicine, Dentistry and Pharmaceutical Sciences, Okayama, Japan

**Keywords:** Cancer-associated fibroblasts, Neutrophil, Anti-fibrotic agent, Pirfenidone

## Abstract

**Background:**

The mechanisms by which neutrophils acquire pro-tumor properties remain poorly understood. In pancreatic cancer, cancer-associated fibroblasts (CAFs) may interact with neutrophils, directing them to promote tumor progression.

**Methods:**

To validate the association between CAFs and neutrophils, the localization of neutrophils was examined in clinically resected pancreatic cancer specimens. CAFs were produced by culturing in cancer-conditioned media, and the effects of these CAFs on neutrophils were examined. In vitro migration and invasion assays assess the effect of CAF-activated neutrophils on cancer cells. The factors secreted by the activated neutrophils were also explored. Finally, pirfenidone (PFD) was tested to determine whether it could suppress the pro-tumor functions of activated neutrophils.

**Results:**

In pancreatic cancer specimens, neutrophils tended to co-localize with IL-6-positive CAFs. Neutrophils co-cultured with CAFs increased migratory capacity and prolonged life span. CAF-affected neutrophils enhance the migratory and invasive activities of pancreatic cancer cells. IL-8 is the most upregulated cytokine secreted by the neutrophils. PFD suppresses IL-8 secretion from CAF-stimulated neutrophils and mitigates the malignant traits of pancreatic cancer cells.

**Conclusion:**

CAFs activate neutrophils and enhance the malignant phenotype of pancreatic cancer. The interactions between cancer cells, CAFs, and neutrophils can be disrupted by PFD, highlighting a potential therapeutic approach.

**Supplementary Information:**

The online version contains supplementary material available at 10.1007/s00262-025-03946-z.

## Introduction

Pancreatic cancer is characterized by desmoplasia as a histopathological feature [1], and the tumor microenvironment (TME) is believed to contribute to its biological malignancy [2, 3]. Cancer cells interact with surrounding cells to form TME [4–7], which is essential for pancreatic cancer [8]. Among the components of the TME, cancer-associated fibroblasts (CAFs) are considered crucial [9], and various mechanisms through which they influence tumor biology have been proposed [10, 11]. However, a comprehensive understanding of the TME is still lacking, and the relationship between CAF and other components of the TME remains unclear [12].

Neutrophils are a significant component of TME [13], but their role has not been well studied owing to their short life span [14, 15]. Recent studies have revealed that neutrophils play a variable role within the TME [16] and significantly impact tumor progression and treatment outcomes [17–19]. Neutrophils secrete various factors and interact with other TME components [20]. Reports are emerging on the interactions between tumors and neutrophils [21] as well as between CAFs and neutrophils [22]. The role of neutrophils influenced by CAFs in tumor dynamics is gaining attention [23], and many aspects remain unknown and require further investigation.

We hypothesized that CAF-influenced neutrophils may be involved in promoting the malignancy of pancreatic cancer cells and investigated the interaction between tumors, CAF, and neutrophils. Our findings indicate that CAF-activated neutrophils enhance the malignant phenotype of cancer cells. Furthermore, pirfenidone (PFD), a drug used to treat idiopathic pulmonary fibrosis, suppresses CAF-induced activation of neutrophils, thereby reducing the malignant traits of pancreatic cancer cells.

## Material and methods

### Cell culture and reagents

The human pancreatic cancer cell lines, MIA PaCa-2 and KP4, were obtained from the American type culture collection. The human fibroblast cell line WI38 was obtained from the Japanese Collection of Research Bioresources. The cells were cultured in RPMI-1640, DMEM, or EMEM supplemented with 10% fetal bovine serum (FBS), 100 U/mL penicillin, and 100 μg/mL streptomycin (P/S) (168–23,191 FUJIFILM). The cultures were maintained at 37 °C in a humidified incubator with 20% O_2_ and 5% CO_2_.

### Isolation of neutrophils

Neutrophils were isolated from healthy volunteers. Whole blood was collected by venipuncture into blood collection tubes coated with EDTA 2 K (#365,900; Becton, Dickinson and Company). Five milliliters of whole blood was layered over 5 mL of Polymorphprep (#114,683, Abbott Diagnostics Technologies AS) in a 15-mL tube and centrifuged at 500 g for 35 min at room temperature. The lower leukocyte band, containing neutrophils, was collected and washed twice with PBS. Neutrophils were resuspended in RPMI-1640 medium without FBS.

### Conditioned media preparation

Conditioned media (CM) from MIA PaCa-2 (MIA PaCa-2-CM) or KP4 (KP4-CM) cells was collected after 48 h of incubation in serum-free RPMI-1640 at 100% confluence. After centrifugation, the supernatant was used as the cancer CM and stored at − 80 °C. Conditioned media from CAFs (CAF-CM) was prepared in the same manner.

### Generation of CAF

WI38 cells were cultured until they reached confluency. The culture medium was then changed to a cancer-conditioned medium (CM) with 10% FBS, and the medium was replaced daily for 4 days. The resulting WI38 cells were designated CAFs.

### Immunofluorescence staining

Paraffin-embedded tissue samples were deparaffinised and rehydrated. Non-specific binding was blocked using 10% donkey serum (Abcam, ab7475) for 30 min at room temperature. To detect neutrophils, iCAFs, and myCAFs, the samples were incubated overnight at 4 °C with anti-myeloperoxidase (MPO) (1:500, RSD, AF3667), anti-IL-6 (1:100, Abcam, ab6672), and anti-αSMA (1:320, Cell Signaling, D4K9N) antibodies, respectively. The tissues were then incubated with secondary antibodies, Alexa Fluor 488 (1:1000, Abcam, ab150129) and Alexa Fluor 647 (1:1000, Abcam, ab150075), in 2% FBS for 1 h at room temperature. Images were obtained using an IX83 microscope (Olympus).

### Flow cytometry

The antibodies (Abs) used for flow cytometry were anti-IL-8, anti-αSMA (Cell Signaling Technology), and anti-rabbit IgG Alexa Fluor 647 (Abcam). Cultured cells were collected, washed twice, and resuspended in 100 µL PBS containing 2% FBS. The cells were fixed in 4% paraformaldehyde solution and stained with specific antibodies. Analyses were performed using a FACScan flow cytometer and the CellQuest software (BD Biosciences).

### Cancer cells migration and invasion assay

In vitro migration and invasion assays were performed in chambers of 8-μm transwell inserts with or without Matrigel (BD FalconTM), respectively. After trypsinization and centrifugation, the cancer cells were placed in the upper chamber of each well insert with serum-free medium, and RPMI-1640 was added to the lower chamber. After incubation for 24 h at 37 °C, the non-invading cancer cells were wiped off, and the cells on the bottom side of the upper chamber were fixed with 4% paraformaldehyde and stained with 0.5% crystal violet. Stained cells were counted under a light microscope at × 200.

### Neutrophil migration assay

The in vitro migration assay was performed using 3-μm Transwell inserts (BD Falcon™). Isolated neutrophils labeled with CellTracker Red (1 µM, Thermo Fisher) were added to serum-free medium in the upper chamber of each insert. Induction factors were added to the lower chamber. After a 3-h incubation at 37 °C, the number of cells that migrated to the lower chamber was determined using an IX83 microscope (Olympus, Tokyo, Japan) at a magnification of × 200.

### Time lapse confocal microscopy

For all video microscopy experiments, time lapse videos were captured using an FV10i confocal microscope (Olympus) equipped with a cell culture chamber. For the neutrophil life span assay, neutrophils were stained with CellTracker Red (1 µM, Thermo Fisher Scientific) and incubated with 10% FBS for 24 h. SYTOX Green (1 µg/mL, Thermo Fisher Scientific) was added to the medium with or without CAF-CM. Time lapse videos were recorded every 30 min for 24 h. The areas of the stained neutrophils were measured.

### Cytokine array

To detect 42 human cytokines in neutrophils, culture supernatants were assayed using a human cytokine antibody array membrane (Abcam, ab133997), according to the manufacturer’s instructions.

### Cell Proliferation assay

MIA PaCa-2 cells were cultured in 24-well plates, and the culture medium was changed (RPMI-1640, non-stimulated neutrophil secretion, CAF-CM-stimulated neutrophil secretion, and CAF-CM-stimulated neutrophil secretion + PFD) after 24 h. After 24 and 48 h, the cells were harvested using trypsin and counted using a cell counter.

### ELISA assay

To evaluate IL-8 levels in neutrophil secretions, culture supernatants were assayed using a human IL-8 enzyme-linked immunosorbent assay (ELISA) kit (Proteintech, KE00006) according to the manufacturer’s instructions.

### Statistical analysis

All statistical analyses were performed using the GraphPad Prism 10 software. The significance of differences between groups was analyzed using Student’s t-test or one-way ANOVA. Statistical significance was set at *P* < 0.05. All experiments were repeated at least thrice.

## Result

### Neutrophils are present with iCAF in the TME of pancreatic cancer

**Fig. 1:**
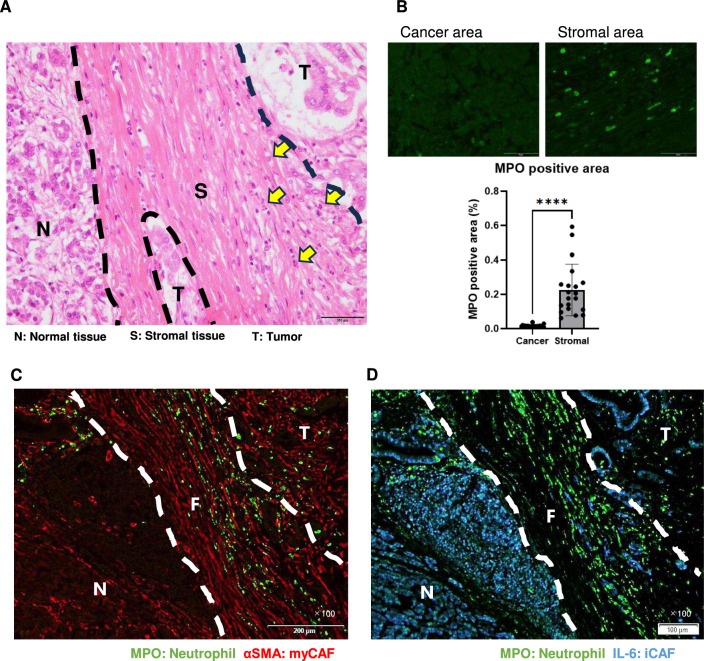
Neutrophils are present with iCAFs in the TME of pancreatic cancer. **A** HE staining of human pancreatic cancer specimens shows neutrophils predominantly in stromal tissue areas. Yellow arrows indicate neutrophils. T: Tumor, S: Stromal tissue, N: Normal tissue. Image taken at 100 × magnification. **B** Comparison of neutrophil presence in cancer cell areas and stromal tissue. Neutrophils were stained with an anti-MPO antibody using fluorescent immunostaining. MPO-positive areas (%) in 20 random fields were compared between cancer cell and stromal tissue areas. Green: MPO-positive neutrophils. Mean ± SD is shown. Each dot represents the MPO-positive area (%) per field (× 400). Student’s t-test was used. ****, *P* < 0.0001. **C** Representative immunofluorescent images showing neutrophils (MPO) and myCAFs (αSMA) in a human pancreatic cancer specimen. Green: Neutrophils; Red: myCAFs. T: Tumor, S: Stromal tissue, N: Normal tissue. Image taken at 100 × magnification. **D** Representative immunofluorescent images showing neutrophils (MPO) and iCAFs (IL-6) in a human pancreatic cancer specimen. Neutrophils tend to co-localize with iCAFs in the stroma. Green: Neutrophils; Blue: iCAFs. T: Tumor, S: Stromal tissue, N: Normal tissue. Image taken at 100 × magnification

To investigate the presence and localization of neutrophils in the TME of pancreatic cancer, clinical specimens were observed with HE staining, revealing neutrophils with segmented nuclei. Neutrophils were more ubiquitously distributed in the stroma than in cancer cell nests (Fig. 1A). Using fluorescent immunostaining of MPO-positive neutrophils, their localization in cancer cells and stromal tissue areas was compared. Neutrophils were significantly more abundant in the stromal areas than in the cancerous areas (Fig. 1B). These findings indicate that neutrophils are present in pancreatic cancer tissues but tend to localize to fibroblasts rather than cancer cells.

The stromal tissue of pancreatic cancer is predominantly formed of fibroblast [24], and recent studies have proposed that fibroblasts can be categorized into myofibroblasts (myCAFs) and inflammatory fibroblasts (iCAFs) [25–27]. It has been reported that myCAFs are present in direct proximity to cancer cells, whereas iCAFs are located more distantly from cancer cells within the dense tumor stroma [26]. To investigate which type of fibroblasts co-localized with neutrophils, fibroblasts were labeled with αSMA, a marker of myCAFs, and IL-6, a marker for iCAFs, and the localization of neutrophils was examined in pancreatic cancer specimens. αSMA-positive myCAFs tended to be located near the cancer region, whereas IL-6-positive iCAFs tended to be distant from the cancer region (Fig. 1C, D). Interestingly, neutrophils identified as MPO-positive cells tended to co-localize more prominently with iCAFs than with myCAFs (Fig. 1C, D).

### iCAFs activate neutrophils and prolong their life span

**Fig. 2:**
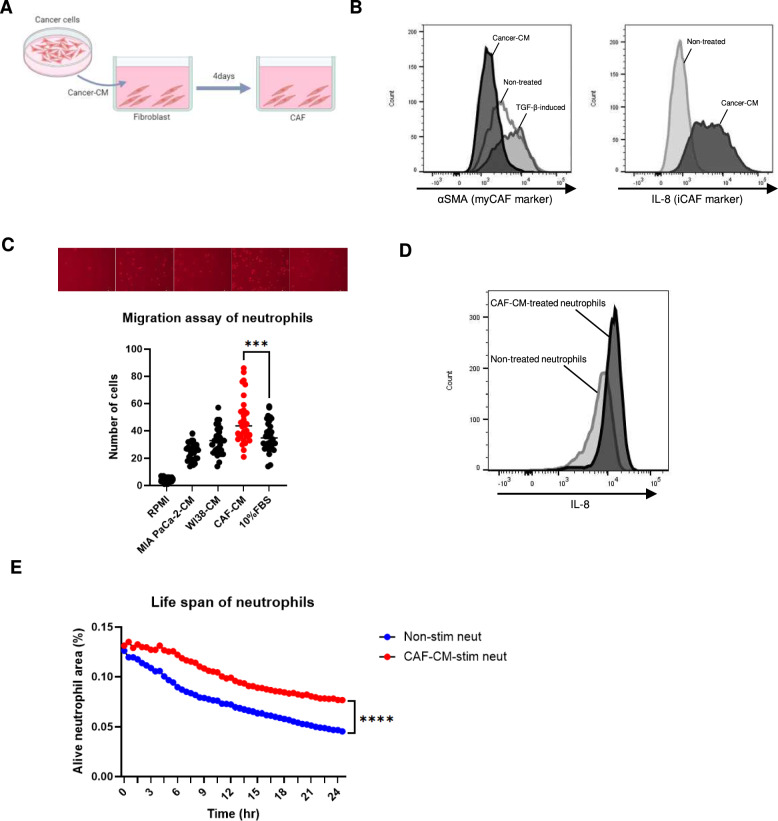
iCAFs increase the migratory ability of neutrophils, activating them, and prolonging their life span. **A** WI38 cells were cultured in cancer CM for 4 days to produce CAFs. **B** *α*SMA and IL-8 are representative markers of myCAF and iCAF, respectively. Left: *α*SMA expression within CAFs induced by MIA PaCa-2-CM was lower than TGF-β-treated CAFs and non-treated control WI38 cells. Right: IL-8 expression within MIA PaCa-2-CM-induced CAFs was higher than in non-treated WI38 cells. **C** Migration assay of neutrophils. Image taken at 100 × magnification and the number of migrated cells per field was counted. Neutrophil migration was significantly increased in the CAF-CM group compared to the FBS-treated positive control. Mean ± SD is shown. Each dot represents the number of cells per field (× 100). One-way ANOVA with Tukey’s test was used. ***, *P* = 0.0004. **D** Compared to non-stimulated neutrophils, those treated with CAF-CM for 24 h showed increased intracellular IL-8. E. Comparing the area of living neutrophils in 9 random fields of view after 24 h, CAF-CM-stimulated neutrophils had a significantly larger area. Student’s t-test was used. ****, *P* < 0.0001

To examine the effect of CAFs on neutrophils in vitro, WI38 human fibroblasts were changed into CAFs using conditioned medium (CM) from MIA PaCa-2 human pancreatic cancer cells (Fig. 2A). WI38 cells were cultured in MIA PaCa-2-CM for four days and then subjected to FACS analysis to assess the expression of *α*SMA, a marker for myCAFs, and IL-8, a marker for iCAFs [28]. The results showed that CAFs induced by MIA PaCa-2-CM were *α*SMA-negative and IL-8-positive, indicating that these fibroblasts had more iCAF properties (Fig. 2B). Similarly, treatment of WI38 cells with KP4-CM induced IL-8-positive iCAFs (Fig. S1).

We hypothesized that CAFs induced by pancreatic cancer cells might affect the phenotype of neutrophils. To assess the effect of CM from CAFs (CAF-CM) on neutrophils, we first investigated whether CAF-CM enhances the migratory ability of neutrophils. Compared with MIA PaCa-2-CM and unstimulated WI38-CM, MIA PaCa-2-induced CAF-CM significantly enhanced neutrophil migration (Fig. 2C). Since IL-8 expression is a marker of neutrophil activation, we investigated whether neutrophils were activated by treatment with CAF-CM by examining intracellular IL-8 in neutrophils using FACS. Compared to untreated neutrophils, those treated with MIA PaCa-2-CAF-CM for 24 h showed elevated IL-8 levels (Fig. 2D). Next, we investigated whether CAF-CM affected the life span of neutrophils. Time lapse observations of MIA PaCa-2-CAF-CM-stimulated neutrophils (CAF-CM-stim neut) showed a significantly longer life span than that of non-stimulated neutrophils (non-stim neut) (Fig. 2E, Supplementary video). These results indicated that CAFs activate neutrophils and prolong their life span.

### Neutrophils stimulated by CAF-CM increase IL-8 secretion, enhancing the malignant trait of cancer cells

**Fig. 3:**
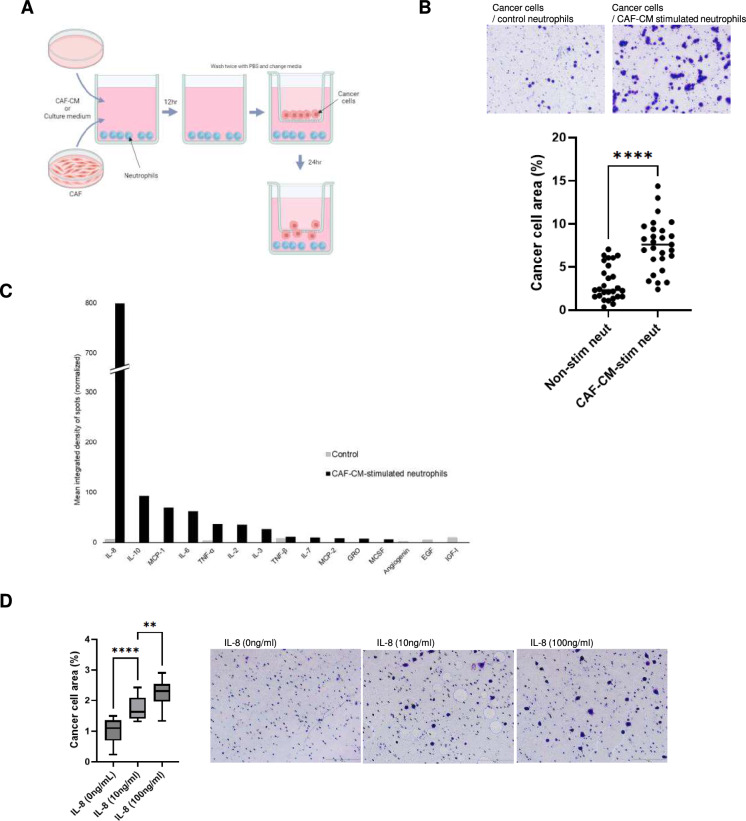
IL-8 is secreted from CAF-CM-activated neutrophils. **A** Schema of the migration and invasion assays of human pancreatic cancer cells co-cultured with neutrophils. Migration and invasion assays of cancer cells were performed using neutrophils as inducers. These neutrophils were cultured in either culture medium or CAF-CM for 12 h and then washed twice with PBS before the assay. **B** CAF-CM-stimulated neutrophils significantly enhanced the migration of cancer cells compared to non-stimulated neutrophils. Mean ± SD is shown. Each dot represents the cancer cell area (%) per field (× 100). Student’s t-test was used. ****, *P* < 0.0001. **C** Cytokine array of neutrophil secretions. Elevated levels of IL-8, IL-10, IL-6, IL-7, and MCP-1 were found in the supernatants of CAF-CM-stimulated neutrophils, with IL-8 being the most increased. **D** Migration assay of MIA PaCa-2 cells using IL-8 as an inducer. Cancer cell migration was enhanced in a concentration-dependent manner by IL-8. Mean ± SD is shown. Each dot represents the number of cells per field (× 100). One-way ANOVA with Tukey’s test was used. **, *P* < 0.005, ***, *P* = 0.0004

To investigate whether CAF-CM-stimulated neutrophils affected the malignant traits of cancer cells, their effects on cancer cell migration and invasion were examined using migration and invasion assays (Fig. 3A). Both the migration and invasion of pancreatic cancer cells were significantly enhanced when co-cultured with neutrophils stimulated by CAF-CM, compared to untreated neutrophils (Fig. 3B).

To identify factors that enhance the migration and invasion of pancreatic cancer cells, secreted factors from neutrophils were analyzed using cytokine arrays (Fig. 3C, S2). Among these, IL-8 was the most significantly increased cytokine in neutrophils stimulated by CAF-CM compared with that in untreated neutrophils (Fig. 3C). IL-8 enhanced the migration and invasion of pancreatic cancer cells, and the migratory ability of these cells was potentiated in an IL-8 concentration-dependent manner (Fig. 3D, S3A). Additionally, IL-8, a chemokine for neutrophils [29], significantly increased the migratory ability of neutrophils (Fig. S3B).

Thus, the results indicate that neutrophils stimulated by CAF-CM increased IL-8 secretion, thereby enhancing the malignant traits of pancreatic cancer cells.

### PFD suppresses IL-8 secretion from neutrophils, thereby suppressing the malignant traits of cancer cells enhanced by neutrophils

As CAFs and neutrophils cooperate to increase cancer malignancy, targeting CAFs could break this malignant cycle. The anti-fibrotic agent pirfenidone (PFD) was tested to determine its effect on tumor-promoting interactions between CAFs, neutrophils, and cancer cells. PFD itself was not cytotoxic to neutrophils and did not alter their morphology (Fig. S4A). Furthermore, PFD did not inhibit the induction of CAFs, as the expression of IL-8 in CAFs was unaffected by PFD treatment during CAF induction by pancreatic cancer CM (Fig. S4B).

**Fig. 4:**
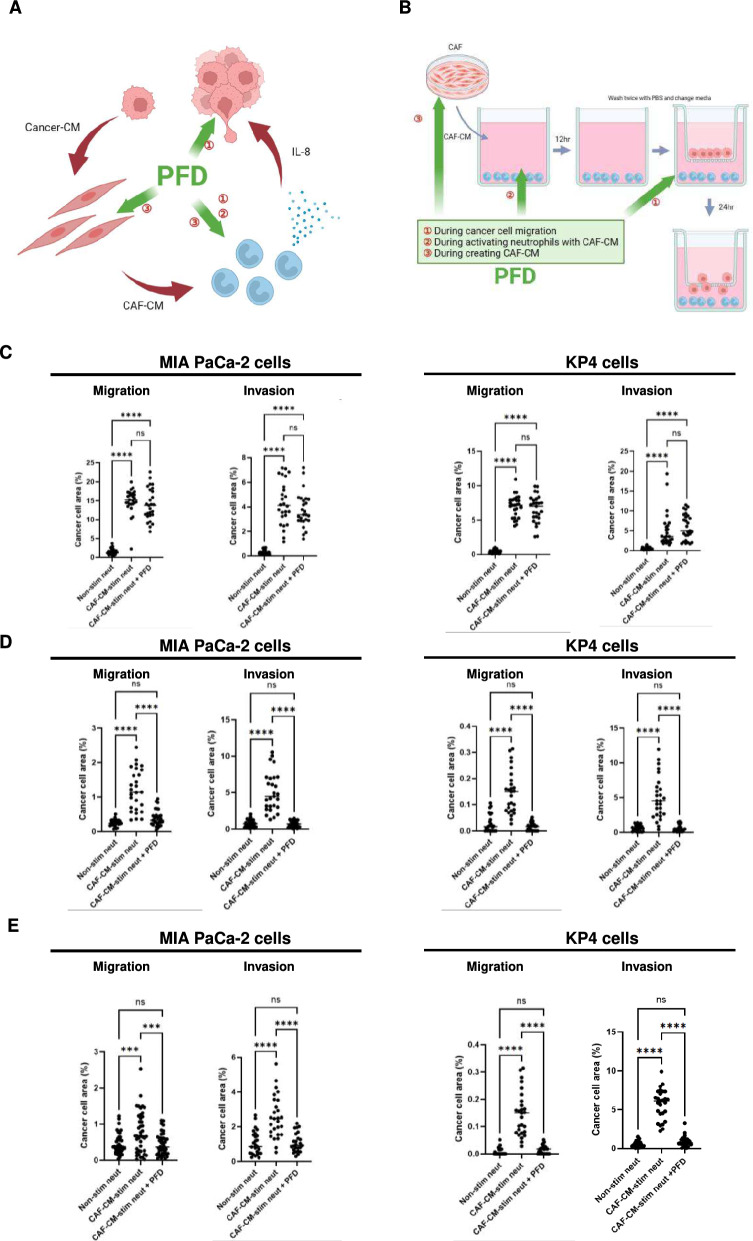
Migration and invasion of human pancreatic cancer cells are enhanced by neutrophils, which are inhibited by PFD. **A** PFD was administered at three different points in the migration or invasion assay of cancer cells using neutrophils as inducers: during cancer cell migration or invasion, during neutrophil activation with CAF-CM, and during CAF-CM creation. **B** Schema of experimental procedure. **C** Administration of PFD during pancreatic cancer cell migration and invasion did not suppress the increased migration and invasion induced by CAF-CM-stimulated neutrophils. Mean ± SD is shown. Each dot represents the cancer cell area (%) per field (× 100). One-way ANOVA with Tukey’s test was used. ****, *P* < 0.0001. **D** Administration of PFD during neutrophil activation with CAF-CM suppressed the increased migration and invasion of cancer cells induced by CAF-CM-stimulated neutrophils. Mean ± SD is shown. Each dot represents the cancer cell area (%) per field (× 100). One-way ANOVA with Tukey’s test was used. ****, *P* < 0.0001. **E** Administration of PFD during CAF-CM creation from CAFs suppressed the increased migration and invasion of cancer cells by CAF-CM-stimulated neutrophils. Mean ± SD is shown. Each dot represents the cancer cell area (%) per field (× 100). One-way ANOVA with Tukey’s test was used. ***, *P* < 0.001, ****, *P* < 0.0001

Next, we investigated whether PFD suppressed the migration and invasion of pancreatic cancer cells promoted by activated neutrophils (Fig. 4A, B). When PFD was added after neutrophil activation, the migration and invasion of pancreatic cancer cells were not suppressed (Fig. 4C). However, when PFD was administered during neutrophil activation, migration and invasion of pancreatic cancer cells were significantly suppressed (Fig. 4D). Furthermore, when PFD was added during CAF-CM production, the ability of CAF-CM to promote neutrophil-mediated migration and invasion of pancreatic cancer cells was abolished (Fig. 4E).

**Fig. 5:**
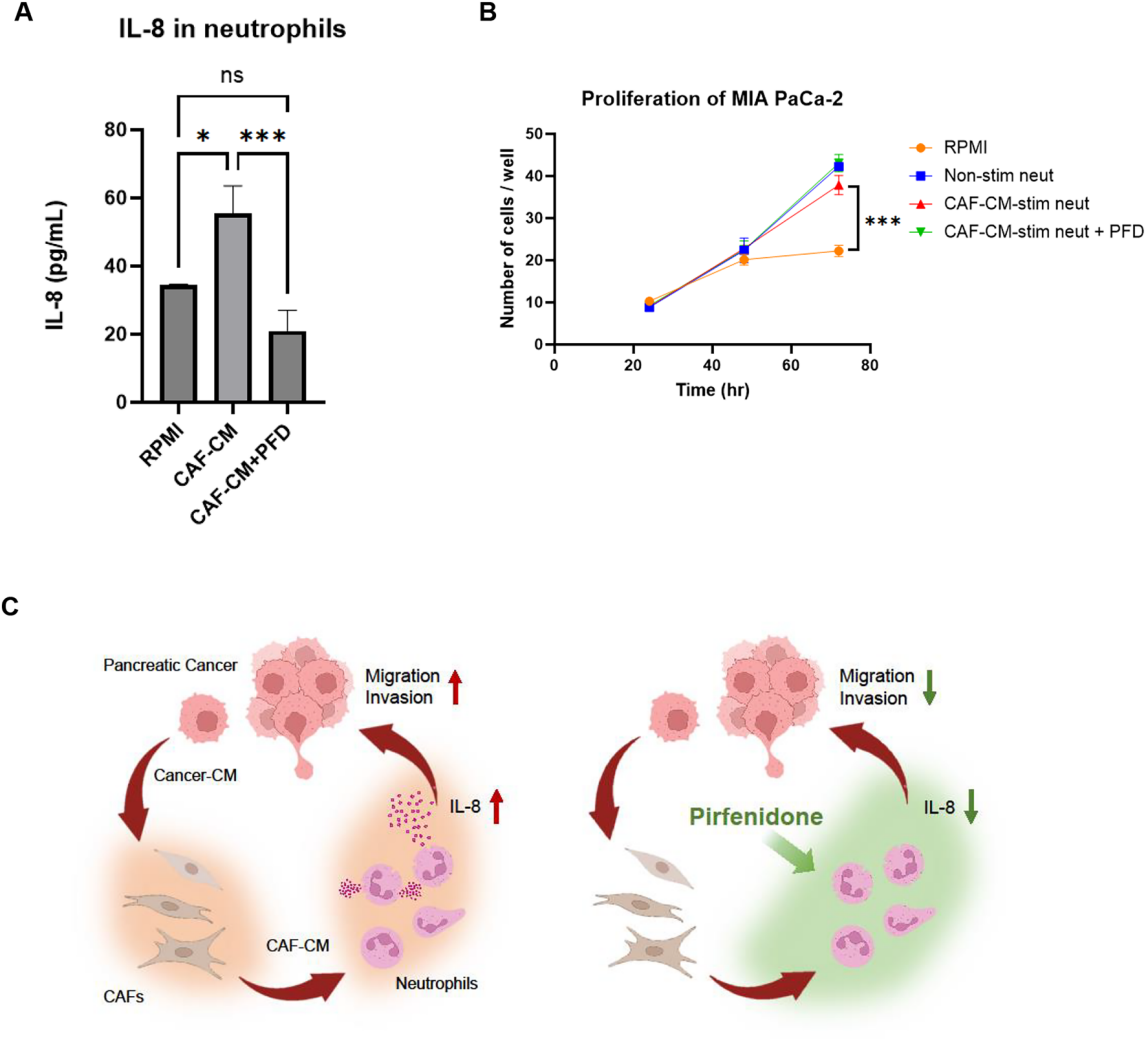
IL-8 secreted by activated neutrophils is suppressed by PFD. **A** IL-8 secretion by neutrophils measured by ELISA. IL-8 secretion was significantly increased in neutrophils stimulated with CAF-CM compared to non-stimulated neutrophils. PFD administration during neutrophil activation with CAF-CM significantly suppressed IL-8 secretion. **B** Effect of secretions from activated neutrophils on cancer cell proliferation. The presence of neutrophils significantly increased MIA PaCa-2 proliferation. Mean ± SD is shown. Each dot represents the number of cells per well. Student’s t-test was used. ***, *P* < 0.001. **C** Pancreatic cancer cells induce fibroblasts to become iCAFs, and iCAF-CM activates neutrophils, increasing their IL-8 secretion and enhancing the malignant traits of pancreatic cancer cells. Conversely, PFD acts on activated neutrophils, suppressing the malignant traits of pancreatic cancer cells. This figure was created with BioRender.com

Although IL-8 levels in neutrophil secretions were elevated by CAF-CM stimulation, PFD administration during neutrophil activation significantly decreased IL-8 secretion by neutrophils (Fig. 5A). Although the presence of neutrophils significantly increased MIA PaCa-2 cell proliferation (Fig. 5B), PFD did not suppress this neutrophil-promoted cancer cell proliferation, indicating that PFD does not directly affect cancer cell growth.

These results indicated that PFD can act at the point where neutrophils are stimulated by CAF-CM, suppressing the production of IL-8 by neutrophils. Therefore, PFD potentially suppresses the malignant traits of pancreatic cancer cells by targeting the CAF–neutrophil axis.

## Discussion

This study investigated how CAF-stimulated neutrophils affect cancer cells and demonstrated that they enhance the malignant traits of cancer cells by increasing IL-8 secretion. In addition, PFD suppressed IL-8 secretion from neutrophils, thereby mitigating the malignant traits of cancer cells (Fig. 5C).

Neutrophil infiltration in various cancer tissues has recently been reported to be associated with a poor prognosis [30], including pancreatic cancer [31, 32]. Clinically, the neutrophil-to-lymphocyte ratio (NLR) has been proposed as a prognostic marker for many cancers [33–35]. The role and importance of neutrophils in cancer have become increasingly apparent, supporting the notion that neutrophils promote cancer progression, although not universally [36]. Neutrophils in cancer retain functional plasticity and can undergo alternative activation when exposed to various cues in the TME [37, 38]. Thus, differences in the localization of neutrophils within tumors may alter their function and clinical implications [36]. However, the mechanisms by which neutrophils are reprogrammed into a tumor-promoting phenotype are not yet clear, and are likely to vary among different cancers.

CAFs are classified into iCAFs and myCAFs, with myCAFs located near the tumor and iCAFs in the surrounding outer area [26]. As previously reported, myCAFs were observed in the proximity of the tumor, and iCAFs were also observed in the outer surrounding area. Interestingly, neutrophils in the TME tended to co-localize with iCAFs. As for the markers for iCAFs, Ohland et al. used IL-6 as a marker for iCAFs when they first defined those distinct fibroblast populations in pancreatic cancer [26]. Subsequently, single-cell analysis of pancreatic ductal adenocarcinoma by Elyada et al. in the same group revealed that iCAFs also express IL-8 [28]. Therefore, IL-8 was adopted as an alternative marker for iCAFs in flow cytometry. Regarding the association between CAFs and neutrophils, this study showed that CAFs enhance neutrophil migration and that IL-8 is a chemokine for neutrophils [19]. Additionally, our data showed that CAFs prolong the life span of neutrophils, which would otherwise be short-lived [39, 40]. Based on these findings, IL-8-expressing iCAFs may attract more neutrophils than myCAFs, and these activated neutrophils can survive longer, resulting in the co-localization of neutrophils with iCAFs in the TME. Other researchers have also reported that CAFs enhance neutrophil migration, suppress apoptosis, and activate neutrophils [23]. Neutrophils tend to coexist more with CAFs in cancer tissues, creating an environment where they can cooperatively influence cancer cells.

There are few reports on the function of CAF-affected neutrophils in cancer cells [22]. Our findings indicate that CAF-affected neutrophils enhance the malignancy of cancer cells. Each component of the cancer microenvironment is thought to be intricately intertwined, and numerous studies have shown the pro-tumor effects of CAFs [41]. Our data provide a novel insight that neutrophils affected by CAFs also directly exert pro-tumor effects on cancer cells. Further research is necessary to elucidate the precise mechanisms by which neutrophils affected by CAFs act on cancer cells and the action of CAFs on neutrophils.

To explore strategies for suppressing the pro-tumor effects of activated neutrophils, we examined the effect of PFD on neutrophils. Although PFD has already been clinically applied for treating idiopathic pulmonary fibrosis [42], it has been reported to inhibit desmoplasia in pancreatic cancer in preclinical models [26, 43]. Furthermore, neutrophils have been shown to increase IL-8 secretion when stimulated with LPS and PFD can suppress IL-8 secretion by neutrophils [44]. These data support our results that administering PFD to neutrophils activated by CAF-CM suppresses IL-8 secretion from neutrophils. Although the detailed mechanism has not been verified, it has been shown that in a rat model of prostatitis, PFD downregulated IL-8 by suppressing the phosphorylation of NF-κB [45]. Further research is needed to understand the mechanism by which PFD suppressed IL-8 secretion by neutrophils in our models.

A limitation of this study is the use of WI38 cells, which are fibroblasts derived from human fetal lung tissue rather than from pancreatic cancer. As such, these cells may not fully represent the CAFs present in the pancreatic tumor microenvironment. Therefore, the interpretation of the results presented in this study should consider this limitation.

Although our in vitro study demonstrated that targeting the CAF–neutrophil axis may have implications for cancer therapy, the feasibility of this approach in vivo remains to be verified. It is also possible that similar mechanisms operate in other tumor microenvironments beyond pancreatic cancer. Extending this research to other cancer types may reveal new therapeutic strategies applicable to various malignancies.

In conclusion, our results revealed the function of CAF-affected neutrophils in cancer cells within the cancer microenvironment, which has been largely unexplored. Considering the development of novel strategies to target the cancer microenvironment, anti-fibroblast agents such as PFD could be a potential approach to inhibit the tumor-promoting properties of activated neutrophils via the suppression of CAFs.

## Supplementary Information

Below is the link to the electronic supplementary material.

Supplementary file1 (AVI 24528 KB)

Supplementary file2 (PDF 783 KB)

Supplementary file3 (AVI 24528 KB)

## Data Availability

No datasets were generated or analyzed during the current study.

## Abbreviations

Abs: Antibodies
αSMA: *α*-Smooth muscle actin
CAF: Cancer-associated fibroblast
CM: Conditioned medium
FBS: Fetal bovine serum
iCAF: Inflammatory CAF
IL-6: Interleukin-6
IL-8: Interleukin-8
MPO: Myeloperoxidase
myCAF: Myofibroblastic CAF
NF-κB: Nuclear factor-κB
PFD: Pirfenidone
P/S: 100 U/mL penicillin and 100 μg/mL streptomycin
TME: Tumor microenvironment

## References

[1] Feig C, Gopinathan A, Neesse A, Chan DS, Cook N, Tuveson DA (2012) The pancreas cancer microenvironment. Clin Cancer Res 18:4266–4276. 10.1158/1078-0432.CCR-11-3114 22896693 10.1158/1078-0432.CCR-11-3114 PMC3442232

[2] Hingorani SR (2023) Epithelial and stromal co-evolution and complicity in pancreatic cancer. Nat Rev Cancer 23:57–77. 10.1038/s41568-022-00530-w 36446904 10.1038/s41568-022-00530-w PMC10470605

[3] Thomas D, Radhakrishnan P (2019) Tumor–stromal crosstalk in pancreatic cancer and tissue fibrosis. Mol Cancer 18:14. 10.1186/s12943-018-0927-5 30665410 10.1186/s12943-018-0927-5 PMC6341551

[4] Ito A, Kagawa S, Sakamoto S et al (2021) Extracellular vesicles shed from gastric cancer mediate protumor macrophage differentiation. BMC Cancer 21:102. 10.1186/s12885-021-07816-6 33509150 10.1186/s12885-021-07816-6 PMC7845052

[5] Sakamoto S, Kagawa S, Kuwada K et al (2019) Intraperitoneal cancer-immune microenvironment promotes peritoneal dissemination of gastric cancer. Oncoimmunology 8:e1671760. 10.1080/2162402X.2019.1671760 31741772 10.1080/2162402X.2019.1671760 PMC6844331

[6] Kuwada K, Kagawa S, Yoshida R et al (2018) The epithelial-to-mesenchymal transition induced by tumor-associated macrophages confers chemoresistance in peritoneally disseminated pancreatic cancer. J Exp Clin Cancer Res 37:307. 10.1186/s13046-018-0981-2 30537992 10.1186/s13046-018-0981-2 PMC6288926

[7] Hanahan D, Weinberg RA (2011) Hallmarks of cancer: the next generation. Cell 144:646–674. 10.1016/j.cell.2011.02.013 21376230 10.1016/j.cell.2011.02.013

[8] Cammarota AL, Falco A, Basile A et al (2023) Pancreatic cancer-secreted proteins: targeting their functions in tumor microenvironment. Cancers (Basel). 10.3390/cancers15194825 37835519 10.3390/cancers15194825 PMC10571538

[9] Bhowmick NA, Neilson EG, Moses HL (2004) Stromal fibroblasts in cancer initiation and progression. Nature 432:332–337. 10.1038/nature03096 15549095 10.1038/nature03096 PMC3050735

[10] Li Y, Tazawa H, Nagai Y et al (2024) Senescent fibroblasts potentiate peritoneal metastasis of diffuse-type gastric cancer cells via IL-8-mediated crosstalk. Anticancer Res 44:2497–2509. 10.21873/anticanres.17056 38821603 10.21873/anticanres.17056

[11] Sahai E, Astsaturov I, Cukierman E et al (2020) A framework for advancing our understanding of cancer-associated fibroblasts. Nat Rev Cancer 20:174–186. 10.1038/s41568-019-0238-1 31980749 10.1038/s41568-019-0238-1 PMC7046529

[12] Mao X, Xu J, Wang W et al (2021) Crosstalk between cancer-associated fibroblasts and immune cells in the tumor microenvironment: new findings and future perspectives. Mol Cancer 20:131. 10.1186/s12943-021-01428-1 34635121 10.1186/s12943-021-01428-1 PMC8504100

[13] Hinshaw DC, Shevde LA (2019) The tumor microenvironment innately modulates cancer progression. Cancer Res 79:4557–4566. 10.1158/0008-5472.CAN-18-3962 31350295 10.1158/0008-5472.CAN-18-3962 PMC6744958

[14] Galli SJ, Borregaard N, Wynn TA (2011) Phenotypic and functional plasticity of cells of innate immunity: macrophages, mast cells and neutrophils. Nat Immunol 12:1035–1044. 10.1038/ni.2109 22012443 10.1038/ni.2109 PMC3412172

[15] Lahoz-Beneytez J, Elemans M, Zhang Y, Ahmed R, Salam A, Block M, Niederalt C, Asquith B, Macallan D (2016) Human neutrophil kinetics: modeling of stable isotope labeling data supports short blood neutrophil half-lives. Blood 127:3431–3438. 10.1182/blood-2016-03-700336 27136946 10.1182/blood-2016-03-700336 PMC4929930

[16] Shafqat A, Khan JA, Alkachem AY, Sabur H, Alkattan K, Yaqinuddin A, Sing GK (2023) How neutrophils shape the immune response: reassessing their multifaceted role in health and disease. Int J Mol Sci. 10.3390/ijms242417583 38139412 10.3390/ijms242417583 PMC10744338

[17] Yoshimoto M, Kagawa S, Kajioka H et al (2023) Dual antiplatelet therapy inhibits neutrophil extracellular traps to reduce liver micrometastases of intrahepatic cholangiocarcinoma. Cancer Lett 567:216260. 10.1016/j.canlet.2023.216260 37295551 10.1016/j.canlet.2023.216260

[18] Hedrick CC, Malanchi I (2022) Neutrophils in cancer: heterogeneous and multifaceted. Nat Rev Immunol 22:173–187. 10.1038/s41577-021-00571-6 34230649 10.1038/s41577-021-00571-6

[19] Kajioka H, Kagawa S, Ito A et al (2021) Targeting neutrophil extracellular traps with thrombomodulin prevents pancreatic cancer metastasis. Cancer Lett 497:1–13. 10.1016/j.canlet.2020.10.015 33065249 10.1016/j.canlet.2020.10.015

[20] Que H, Fu Q, Lan T, Tian X, Wei X (2022) Tumor-associated neutrophils and neutrophil-targeted cancer therapies. Biochim Biophys Acta Rev Cancer 1877:188762. 10.1016/j.bbcan.2022.188762 35853517 10.1016/j.bbcan.2022.188762

[21] Sturgeon R, Goel P, Singh RK (2023) Tumor-associated neutrophils in pancreatic cancer progression and metastasis. Am J Cancer Res 13:6176–6189 38187037 PMC10767342

[22] Zhang C, Fei Y, Wang H, Hu S, Liu C, Hu R, Du Q (2023) CAFs orchestrates tumor immune microenvironment-A new target in cancer therapy? Front Pharmacol 14:1113378. 10.3389/fphar.2023.1113378 37007004 10.3389/fphar.2023.1113378 PMC10064291

[23] Cheng Y, Li H, Deng Y, Tai Y, Zeng K, Zhang Y, Liu W, Zhang Q, Yang Y (2018) Cancer-associated fibroblasts induce PDL1+ neutrophils through the IL6-STAT3 pathway that foster immune suppression in hepatocellular carcinoma. Cell Death Dis 9:422. 10.1038/s41419-018-0458-4 29556041 10.1038/s41419-018-0458-4 PMC5859264

[24] Hosein AN, Brekken RA, Maitra A (2020) Pancreatic cancer stroma: an update on therapeutic targeting strategies. Nat Rev Gastroenterol Hepatol 17:487–505. 10.1038/s41575-020-0300-1 32393771 10.1038/s41575-020-0300-1 PMC8284850

[25] Miyazaki Y, Oda T, Mori N, Kida YS (2020) Adipose-derived mesenchymal stem cells differentiate into pancreatic cancer-associated fibroblasts in vitro. FEBS Open Bio 10:2268–2281. 10.1002/2211-5463.12976 32931156 10.1002/2211-5463.12976 PMC7609785

[26] Ohlund D, Handly-Santana A, Biffi G et al (2017) Distinct populations of inflammatory fibroblasts and myofibroblasts in pancreatic cancer. J Exp Med 214:579–596. 10.1084/jem.20162024 28232471 10.1084/jem.20162024 PMC5339682

[27] Piersma B, Hayward MK, Weaver VM (2020) Fibrosis and cancer: a strained relationship. Biochim Biophys Acta Rev Cancer 1873:188356. 10.1016/j.bbcan.2020.188356 32147542 10.1016/j.bbcan.2020.188356 PMC7733542

[28] Elyada E, Bolisetty M, Laise P et al (2019) Cross-species single-cell analysis of pancreatic ductal adenocarcinoma reveals antigen-presenting cancer-associated fibroblasts. Cancer Discov 9:1102–1123. 10.1158/2159-8290.CD-19-0094 31197017 10.1158/2159-8290.CD-19-0094 PMC6727976

[29] Foxman EF, Kunkel EJ, Butcher EC (1999) Integrating conflicting chemotactic signals. The role of memory in leukocyte navigation. J Cell Biol 147:577–588. 10.1083/jcb.147.3.577 10545501 10.1083/jcb.147.3.577 PMC2151176

[30] Gentles AJ, Newman AM, Liu CL et al (2015) The prognostic landscape of genes and infiltrating immune cells across human cancers. Nat Med 21:938–945. 10.1038/nm.3909 26193342 10.1038/nm.3909 PMC4852857

[31] Zhang J, Xu X, Shi M et al (2017) CD13(hi) Neutrophil-like myeloid-derived suppressor cells exert immune suppression through Arginase 1 expression in pancreatic ductal adenocarcinoma. Oncoimmunology 6:e1258504. 10.1080/2162402X.2016.1258504 28344866 10.1080/2162402X.2016.1258504 PMC5353902

[32] Ino Y, Yamazaki-Itoh R, Shimada K, Iwasaki M, Kosuge T, Kanai Y, Hiraoka N (2013) Immune cell infiltration as an indicator of the immune microenvironment of pancreatic cancer. Br J Cancer 108:914–923. 10.1038/bjc.2013.32 23385730 10.1038/bjc.2013.32 PMC3590668

[33] Chen Y, Yan H, Wang Y, Shi Y, Dai G (2017) Significance of baseline and change in neutrophil-to-lymphocyte ratio in predicting prognosis: a retrospective analysis in advanced pancreatic ductal adenocarcinoma. Sci Rep 7:753. 10.1038/s41598-017-00859-5 28392554 10.1038/s41598-017-00859-5 PMC5429710

[34] Ma LX, Wang Y, Espin-Garcia O et al (2023) Systemic inflammatory prognostic scores in advanced pancreatic adenocarcinoma. Br J Cancer 128:1916–1921. 10.1038/s41416-023-02214-0 36927977 10.1038/s41416-023-02214-0 PMC10147590

[35] Templeton AJ, McNamara MG, Šeruga B et al (2014) Prognostic role of neutrophil-to-lymphocyte ratio in solid tumors: a systematic review and meta-analysis. JNCI: J National Cancer Ins. 10.1093/jnci/dju124 10.1093/jnci/dju124 24875653

[36] Shaul ME, Fridlender ZG (2019) Tumour-associated neutrophils in patients with cancer. Nat Rev Clin Oncol 16:601–620. 10.1038/s41571-019-0222-4 31160735 10.1038/s41571-019-0222-4

[37] Fridlender ZG, Sun J, Kim S, Kapoor V, Cheng G, Ling L, Worthen GS, Albelda SM (2009) Polarization of tumor-associated neutrophil phenotype by TGF-beta: “N1” versus “N2” TAN. Cancer Cell 16:183–194. 10.1016/j.ccr.2009.06.017 19732719 10.1016/j.ccr.2009.06.017 PMC2754404

[38] Giese MA, Hind LE, Huttenlocher A (2019) Neutrophil plasticity in the tumor microenvironment. Blood 133:2159–2167. 10.1182/blood-2018-11-844548 30898857 10.1182/blood-2018-11-844548 PMC6524564

[39] Coffelt SB, Wellenstein MD, de Visser KE (2016) Neutrophils in cancer: neutral no more. Nat Rev Cancer 16:431–446. 10.1038/nrc.2016.52 27282249 10.1038/nrc.2016.52

[40] Colotta F, Re F, Polentarutti N, Sozzani S, Mantovani A (1992) Modulation of granulocyte survival and programmed cell death by cytokines and bacterial products. Blood 80:2012–2020 1382715

[41] Caligiuri G, Tuveson DA (2023) Activated fibroblasts in cancer: perspectives and challenges. Cancer Cell 41:434–449. 10.1016/j.ccell.2023.02.015 36917949 10.1016/j.ccell.2023.02.015 PMC11022589

[42] Noble PW, Albera C, Bradford WZ et al (2011) Pirfenidone in patients with idiopathic pulmonary fibrosis (CAPACITY): two randomised trials. Lancet 377:1760–1769. 10.1016/S0140-6736(11)60405-4 21571362 10.1016/S0140-6736(11)60405-4

[43] Kozono S, Ohuchida K, Eguchi D, Ikenaga N, Fujiwara K, Cui L, Mizumoto K, Tanaka M (2013) Pirfenidone inhibits pancreatic cancer desmoplasia by regulating stellate cells. Cancer Res 73:2345–2356. 10.1158/0008-5472.CAN-12-3180 23348422 10.1158/0008-5472.CAN-12-3180

[44] Evani SJ, Karna SLR, Seshu J, Leung KP (2020) Pirfenidone regulates LPS mediated activation of neutrophils. Sci Rep 10:19936. 10.1038/s41598-020-76271-3 33203891 10.1038/s41598-020-76271-3 PMC7672086

[45] Peng X, Guo H, Chen J, Wang J, Huang J (2020) The effect of pirfenidone on rat chronic prostatitis/chronic pelvic pain syndrome and its mechanisms. Prostate 80:917–925. 10.1002/pros.23995 32569423 10.1002/pros.23995

