# Supplementary material for: Cancer-associated fibroblasts promote pro-tumor functions of neutrophils in pancreatic cancer via IL-8: potential suppression by pirfenidone
Source: Cancer Immunol Immunother. 2025 Feb 4;74(3):96. doi: 10.1007/s00262-025-03946-z (PMC11794937; doi:10.1007/s00262-025-03946-z)
Supplement: Supplementary file 2 — Supplementary file2 (PDF 783 KB) [file 262_2025_3946_MOESM2_ESM.pdf]

**Figure S1**

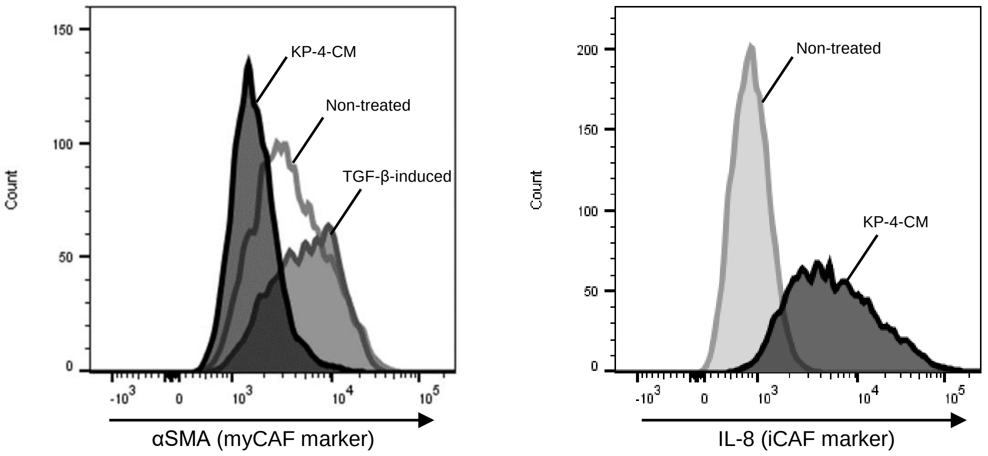


Figure S1
WI38 cells were cultured in KP-4 CM for 4 days to produce CAFs. B. αSMA and IL-8 are representative markers of myCAF and iCAF, respectively. Left: αSMA expression within CAFs induced by KP-4-CM was lower than TGF-β-treated CAFs, and non-treated control WI38 cells. Right: IL-8 expression within KP-4-CM-induced CAFs was higher than non-treated WI38.

# Figure S2

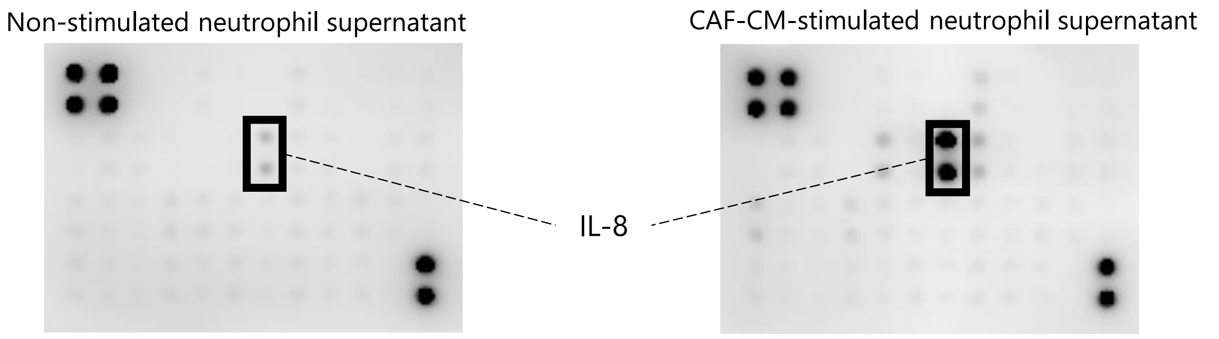


Figure S2.
Cytokine array of secretions from neutrophils.

# Figure S3

A

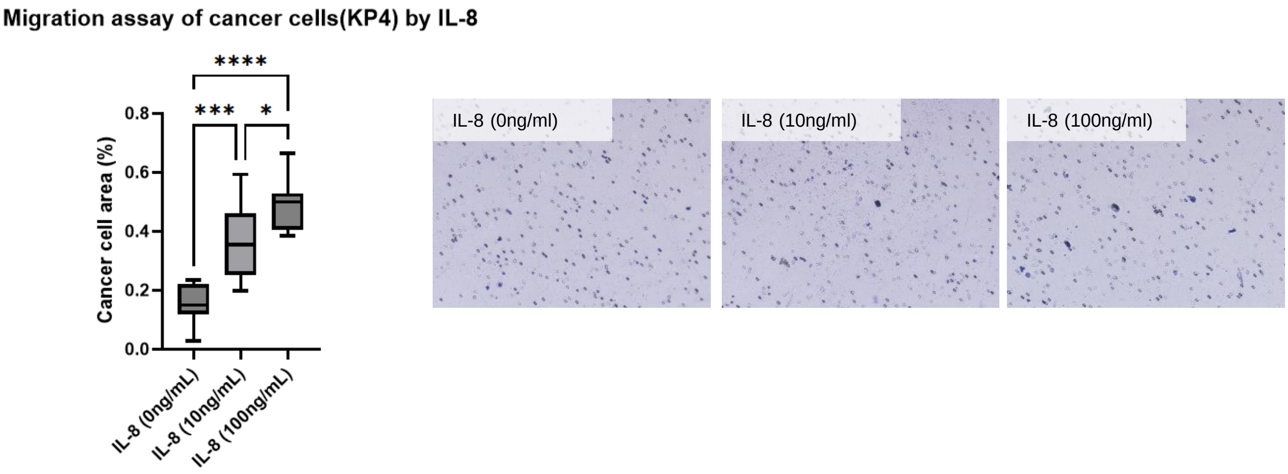


B

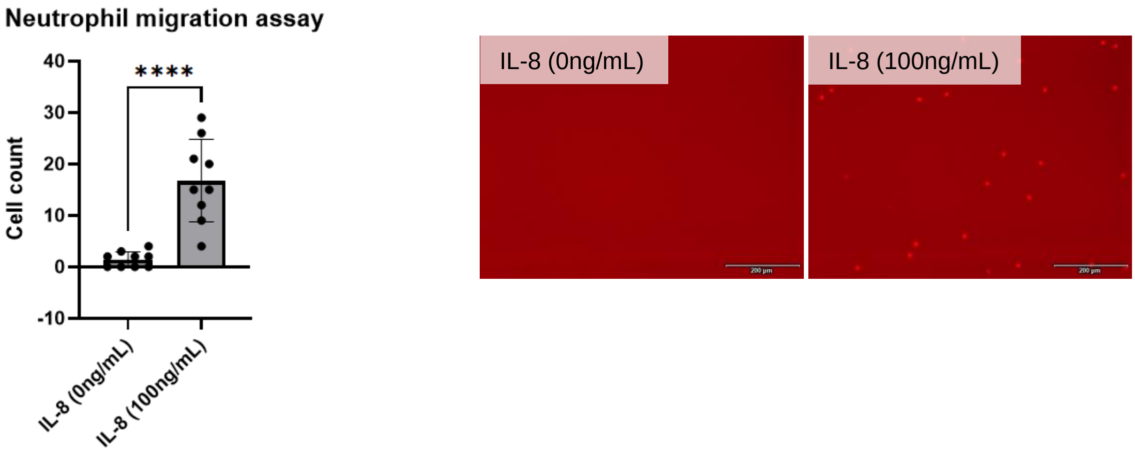


Figure S3
A. Migration assay of cancer cells (MIA PaCa-2) using IL-8 as an inducer. Cancer cell migration was enhanced in a concentration-dependent manner by IL-8. Mean ± SD is shown in each graph. Each dot on the graph represents the number of cells / field (x 100). One-way ANOVA with Tukey’s test was used in the figure.**, P<0.005, ***, P=0.0004.
B. Neutrophil migration assay with or without IL-8. Neutrophils were dyed with CellTracker Red. IL-8 enhances the migratory ability of neutrophils. Mean ± SD is shown in each graph. Each dot on the graph represents the cancer cell area (%) / field (x 100). Student’s t-test was used in the figure. ****, P<0.0001.

# Figure S4

**A**

Non-stim-neut　　CAF-CM-stim-neut　　CAF-CM-stim-neut + PFD

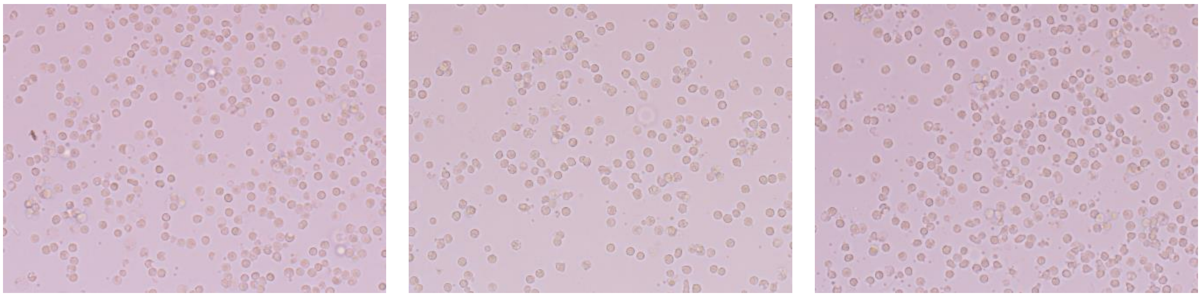

**B**

IL-8 (iCAF marker)　　IL-8 (iCAF marker)

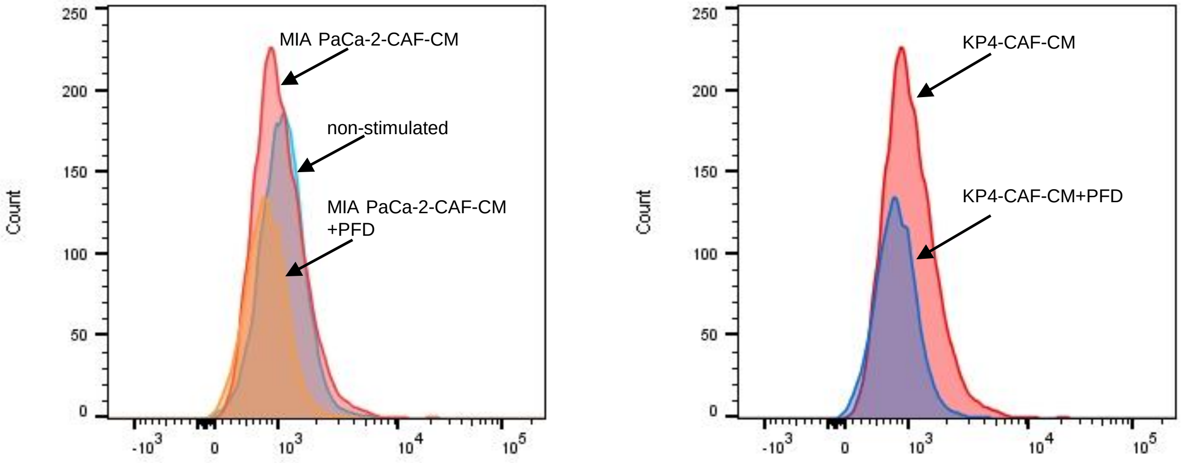


Figure S4

A. Neutrophils 12 hours after each treatment. We did not observe any changes in neutrophil morphology.

B. B. PFD did not inhibit the shift of WI38 to CAF by MIA PaCa-2-CM or KP4-CM.
